# Supplementary material for: HIVprotI: an integrated web based platform for prediction and design of HIV proteins inhibitors
Source: J Cheminform. 2018 Mar 9;10:12. doi: 10.1186/s13321-018-0266-y (PMC5845081; doi:10.1186/s13321-018-0266-y)
Supplement: Supplementary file 2 — Additional file 2. Source code of HIVProtI web server. [file 13321_2018_266_MOESM2_ESM.zip › HIVprotI_Source-code/hivprotI/weka-3-6-6/doc/weka/classifiers/Classifier.html]

Classifier


---


|  |  |  |
| --- | --- | --- |
| **Overview** \| **Package** \| **Class** \| **Tree** \| **Deprecated** \| **Index** \| **Help** \| **Weka's home** | |  |
| **PREV CLASS**   **NEXT CLASS** | **FRAMES**    **NO FRAMES**     **All Classes** |  |
| SUMMARY: NESTED \| FIELD \| CONSTR \| METHOD | DETAIL: FIELD \| CONSTR \| METHOD |  |


---


## weka.classifiers Class Classifier

```
java.lang.Object
  weka.classifiers.Classifier
```

**All Implemented Interfaces:**: java.io.Serializable, java.lang.Cloneable, CapabilitiesHandler, OptionHandler, RevisionHandler

**Direct Known Subclasses:**: ADTree, AODE, AODEsr, BayesianLogisticRegression, BayesNet, CitationKNN, ClassificationViaClustering, ComplementNaiveBayes, ConjunctiveRule, DecisionStump, DecisionTable, DMNBtext, FT, GaussianProcesses, HNB, HyperPipes, IB1, IBk, Id3, IsotonicRegression, J48, J48graft, JRip, KStar, LADTree, LBR, LeastMedSq, LibLINEAR, LibSVM, LinearRegression, LMT, Logistic, LogisticBase, M5Base, MDD, MIDD, MILR, MINND, MIOptimalBall, MISMO, MISVM, MultilayerPerceptron, MultipleClassifiersCombiner, NaiveBayes, NaiveBayesMultinomial, NaiveBayesSimple, NBTree, NNge, OneR, PaceRegression, PART, PLSClassifier, PMMLClassifier, PreConstructedLinearModel, Prism, RandomForest, RandomizableClassifier, RandomTree, RBFNetwork, REPTree, Ridor, RuleNode, SerializedClassifier, SimpleLinearRegression, SimpleLogistic, SingleClassifierEnhancer, SMO, SMOreg, SPegasos, UserClassifier, VFI, VotedPerceptron, WAODE, Winnow, ZeroR

---

``` public abstract class Classifier extends java.lang.Object implements java.lang.Cloneable, java.io.Serializable, OptionHandler, CapabilitiesHandler, RevisionHandler ```

Abstract classifier. All schemes for numeric or nominal prediction in
Weka extend this class. Note that a classifier MUST either implement
distributionForInstance() or classifyInstance().

**Version:**
:   $Revision: 5536 $

**Author:**
:   Eibe Frank (eibe@cs.waikato.ac.nz), Len Trigg (trigg@cs.waikato.ac.nz)

**See Also:**: Serialized Form

---

**Constructor Summary**

`Classifier()`


| **Method Summary** | |
| --- | --- |
| `abstract  void` | `buildClassifier(Instances data)`             Generates a classifier. |
| `double` | `classifyInstance(Instance instance)`             Classifies the given test instance. |
| `java.lang.String` | `debugTipText()`             Returns the tip text for this property |
| `double[]` | `distributionForInstance(Instance instance)`             Predicts the class memberships for a given instance. |
| `static Classifier` | `forName(java.lang.String classifierName, java.lang.String[] options)`             Creates a new instance of a classifier given it's class name and (optional) arguments to pass to it's setOptions method. |
| `Capabilities` | `getCapabilities()`             Returns the Capabilities of this classifier. |
| `boolean` | `getDebug()`             Get whether debugging is turned on. |
| `java.lang.String[]` | `getOptions()`             Gets the current settings of the Classifier. |
| `java.lang.String` | `getRevision()`             Returns the revision string. |
| `java.util.Enumeration` | `listOptions()`             Returns an enumeration describing the available options. |
| `static Classifier[]` | `makeCopies(Classifier model, int num)`             Creates a given number of deep copies of the given classifier using serialization. |
| `static Classifier` | `makeCopy(Classifier model)`             Creates a deep copy of the given classifier using serialization. |
| `void` | `setDebug(boolean debug)`             Set debugging mode. |
| `void` | `setOptions(java.lang.String[] options)`             Parses a given list of options. |

**Methods inherited from class java.lang.Object**

`equals, getClass, hashCode, notify, notifyAll, toString, wait, wait, wait`

**Constructor Detail**

### Classifier

```
public Classifier()
```


**Method Detail**

### buildClassifier

```
public abstract void buildClassifier(Instances data)
                              throws java.lang.Exception
```

:   Generates a classifier. Must initialize all fields of the classifier
    that are not being set via options (ie. multiple calls of buildClassifier
    must always lead to the same result). Must not change the dataset
    in any way.

    :   **Parameters:**: `data` - set of instances serving as training data **Throws:**: `java.lang.Exception` - if the classifier has not been generated successfully

---


### classifyInstance

```
public double classifyInstance(Instance instance)
                        throws java.lang.Exception
```

:   Classifies the given test instance. The instance has to belong to a
    dataset when it's being classified. Note that a classifier MUST
    implement either this or distributionForInstance().

    :   **Parameters:**: `instance` - the instance to be classified **Returns:**: the predicted most likely class for the instance or Instance.missingValue() if no prediction is made **Throws:**: `java.lang.Exception` - if an error occurred during the prediction

---


### distributionForInstance

```
public double[] distributionForInstance(Instance instance)
                                 throws java.lang.Exception
```

:   Predicts the class memberships for a given instance. If
    an instance is unclassified, the returned array elements
    must be all zero. If the class is numeric, the array
    must consist of only one element, which contains the
    predicted value. Note that a classifier MUST implement
    either this or classifyInstance().

    :   **Parameters:**: `instance` - the instance to be classified **Returns:**: an array containing the estimated membership probabilities of the test instance in each class or the numeric prediction **Throws:**: `java.lang.Exception` - if distribution could not be computed successfully

---


### forName

```
public static Classifier forName(java.lang.String classifierName,
                                 java.lang.String[] options)
                          throws java.lang.Exception
```

:   Creates a new instance of a classifier given it's class name and
    (optional) arguments to pass to it's setOptions method. If the
    classifier implements OptionHandler and the options parameter is
    non-null, the classifier will have it's options set.

    :   **Parameters:**: `classifierName` - the fully qualified class name of the classifier: `options` - an array of options suitable for passing to setOptions. May be null. **Returns:**: the newly created classifier, ready for use. **Throws:**: `java.lang.Exception` - if the classifier name is invalid, or the options supplied are not acceptable to the classifier

---


### makeCopy

```
public static Classifier makeCopy(Classifier model)
                           throws java.lang.Exception
```

:   Creates a deep copy of the given classifier using serialization.

    :   **Parameters:**: `model` - the classifier to copy **Returns:**: a deep copy of the classifier **Throws:**: `java.lang.Exception` - if an error occurs

---


### makeCopies

```
public static Classifier[] makeCopies(Classifier model,
                                      int num)
                               throws java.lang.Exception
```

:   Creates a given number of deep copies of the given classifier using serialization.

    :   **Parameters:**: `model` - the classifier to copy: `num` - the number of classifier copies to create. **Returns:**: an array of classifiers. **Throws:**: `java.lang.Exception` - if an error occurs

---


### listOptions

```
public java.util.Enumeration listOptions()
```

:   Returns an enumeration describing the available options.

    :   **Specified by:**: `listOptions` in interface `OptionHandler`
    :   **Returns:**: an enumeration of all the available options.

---


### setOptions

```
public void setOptions(java.lang.String[] options)
                throws java.lang.Exception
```

:   Parses a given list of options. Valid options are:

    -D   
    If set, classifier is run in debug mode and
    may output additional info to the console.

    :   **Specified by:**: `setOptions` in interface `OptionHandler`
    :   **Parameters:**: `options` - the list of options as an array of strings **Throws:**: `java.lang.Exception` - if an option is not supported

---


### getOptions

```
public java.lang.String[] getOptions()
```

:   Gets the current settings of the Classifier.

    :   **Specified by:**: `getOptions` in interface `OptionHandler`
    :   **Returns:**: an array of strings suitable for passing to setOptions

---


### setDebug

```
public void setDebug(boolean debug)
```

:   Set debugging mode.

    :   **Parameters:**: `debug` - true if debug output should be printed

---


### getDebug

```
public boolean getDebug()
```

:   Get whether debugging is turned on.

    :   **Returns:**: true if debugging output is on

---


### debugTipText

```
public java.lang.String debugTipText()
```

:   Returns the tip text for this property

    :   **Returns:**: tip text for this property suitable for displaying in the explorer/experimenter gui

---


### getCapabilities

```
public Capabilities getCapabilities()
```

:   Returns the Capabilities of this classifier. Maximally permissive
    capabilities are allowed by default. Derived classifiers should
    override this method and first disable all capabilities and then
    enable just those capabilities that make sense for the scheme.

    :   **Specified by:**: `getCapabilities` in interface `CapabilitiesHandler`
    :   **Returns:**: the capabilities of this object **See Also:**: `Capabilities`

---


### getRevision

```
public java.lang.String getRevision()
```

:   Returns the revision string.

    :   **Specified by:**: `getRevision` in interface `RevisionHandler`
    :   **Returns:**: the revision


---


|  |  |  |
| --- | --- | --- |
| **Overview** \| **Package** \| **Class** \| **Tree** \| **Deprecated** \| **Index** \| **Help** \| **Weka's home** | |  |
| **PREV CLASS**   **NEXT CLASS** | **FRAMES**    **NO FRAMES**     **All Classes** |  |
| SUMMARY: NESTED \| FIELD \| CONSTR \| METHOD | DETAIL: FIELD \| CONSTR \| METHOD |  |


---
